# Patching Holes in the *Chlamydomonas* Genome

**DOI:** 10.1534/g3.116.029207

**Published:** 2016-05-10

**Authors:** Frej Tulin, Frederick R. Cross

**Affiliations:** The Rockefeller University, New York, New York 10065

**Keywords:** transcriptome assembly, trinity, *Chlamydomonas*

## Abstract

The *Chlamydomonas* genome has been sequenced, assembled, and annotated to produce a rich resource for genetics and molecular biology in this well-studied model organism. However, the current reference genome contains ∼1000 blocks of unknown sequence (‘N-islands’), which are frequently placed in introns of annotated gene models. We developed a strategy to search for previously unknown exons hidden within such blocks, and determine the sequence, and exon/intron boundaries, of such exons. These methods are based on assembly and alignment of short cDNA and genomic DNA reads, completely independent of prior reference assembly or annotation. Our evidence indicates that a substantial proportion of the annotated intronic N-islands contain hidden exons. For most of these, our algorithm recovers full exonic sequence with associated splice junctions and exon-adjacent intronic sequence. These new exons represent *de novo* sequence generally present nowhere in the assembled genome, and the added sequence improves evolutionary conservation of the predicted encoded peptides.

The assembled *Chlamydomonas* reference genome is 120 Mb long, 65% GC, and repeat-rich. The assembly contains 17 chromosomes (∼1–10 Mb) and a further 37 ‘scaffolds’ (0.1–0.8 Mb) (Merchant *et al.* 2007). The reference assembly, and an annotated genome including full transcript models (Blaby *et al.* 2014), is available on the public access Phytozome website (http/phytozome.jgi.doe.gov); we will refer to the sequence assembly and annotations as ‘Phytozome’ or ‘reference.’ About 3.5% of the nucleotides are indicated as ’N’ (unknown sequence). These Ns are distributed in ‘N-islands’ of varying length, mostly ∼100 nt; most islands are annotated as being present within intronic sequence. These islands are effectively ‘placeholders’, where the length of the island is intended to reflect an estimate of the amount of missing sequence (Merchant *et al.* 2007).

Here, we present bioinformatic and experimental methods to determine whether N-islands assigned to introns are entirely intronic, or instead contain exons missing from the assembled sequence. Our methods allow determination of the sequence of these hidden exons along with flanking intronic sequence. While we focus here on the specific set of N-island-containing transcript models in *Chlamydomonas*, the approach can be used more generally to ‘proofread’ transcript models from publically available or *de novo* assembled transcriptomes.

## Materials and Methods

Genomic sequences and transcript models (Merchant *et al.* 2007; Blaby *et al.* 2014) were downloaded from Phytozome. Illumina RNAseq libraries were described in Tulin and Cross (2015). Illumina genomic libraries were from parental strains in the CC124 background used for the RNAseq experiments (Tulin and Cross 2014, 2015). This background is closely related, but not identical, to the genome reference strain, CC-503 (Flowers *et al.* 2015; Gallaher *et al.* 2015). We used these RNAseq libraries because we were especially interested in genes involved in mitotic progression; many such genes are highly enriched for expression in the synchronized libraries we used (Tulin and Cross 2015). Alignment of RNAseq reads to Phytozome transcript models was described (Tulin and Cross 2015). We downloaded BLAST (Altschul *et al.* 1990), Trinity (Grabherr *et al.* 2011; Haas *et al.* 2013), clustalo (Sievers *et al.* 2011), and the Bowtie2 aligner (Langmead and Salzberg 2012). The latter was used in ‘local’ mode to align genomic reads to Trinity objects. Trinity and BLAST results were analyzed with shell scripts and Perl code. Analysis of genomic reads aligned to Trinity objects was carried out with custom MATLAB code.

We used the Trinity software suite (Grabherr *et al.* 2011; Haas *et al.* 2013) to assemble a *de novo* transcriptome for *Chlamydomonas*, using RNAseq reads that we generated from multiple diurnal cell cycle time courses (Tulin and Cross 2015). Since we were primarily interested in evaluating N-islands within gene models, we restricted the RNAseq data input to ∼57 million 50-bp single-end reads that either (1) aligned to a transcript models that contain at least one N-island, or (2) did not align to any transcript model.

To focus on sequences that are likely to contain useful biological information, we filtered the initial 10,134-sequence Trinity output (median sequence length = 315 bp) to retain only sequences that had an e-value (BLASTX) to *Volvox* or *Arabidopsis* (TAIR10) peptides of 0.001 or lower. *Volvox* is a recently diverged multicellular relative of *Chlamydomonas*, with approximately 50% neutral sequence divergence but high conservation of many predicted encoded proteins (Prochnik *et al.* 2010). This reduced the number of sequences to 3473, indicating that many shorter sequences may be either Trinity assembly errors or else too short to generate significant BLASTX alignments. The shortened list contained many similar sequences. To filter out shorter fragments that were potentially partial versions of longer sequences in the set, we used the uclust software (Edgar 2010). This narrowed the result to a final list of 3114 unique Trinity-generated primary sequences (Supplemental Material, File S2), enriched in longer sequences (median length = 446 bp) containing potentially conserved protein coding sequence.

Perl and MATLAB codes and detailed results from the analysis are provided in File S1, File S3, File S5, and File S6.

### Data availability

The authors state that all data necessary for confirming the conclusions presented in the article are represented fully within the article. Complete raw data and accessory files used in the computations are also available on request.

## Results

### Targeted de novo transcriptome assembly

**Figure 1:**
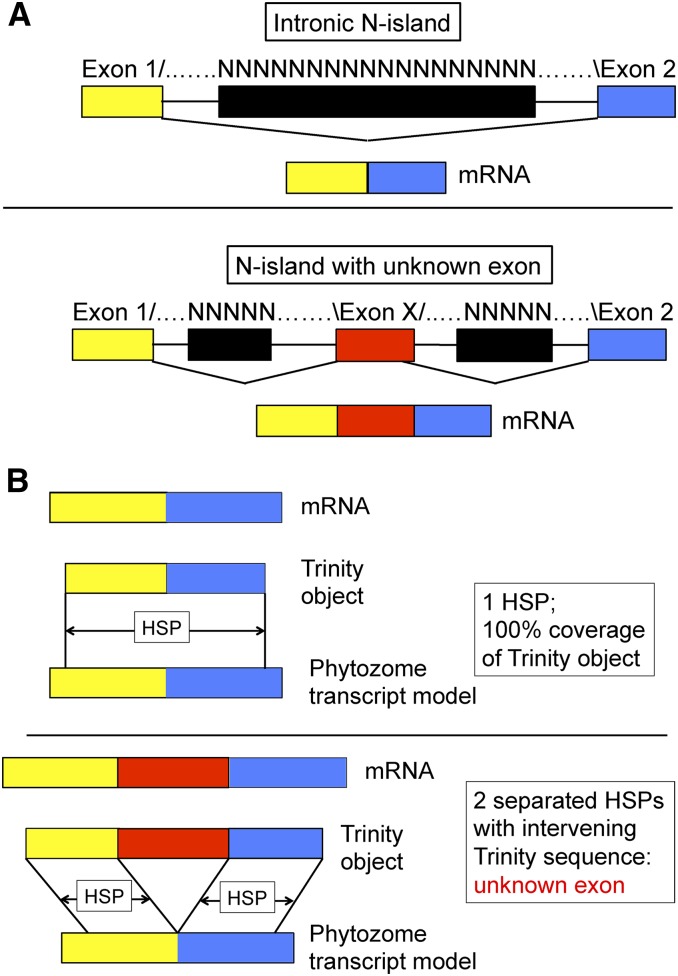
N-islands annotated as intronic can contain hidden exons: the separated high-scoring pair (HSP) test. (A) Top: Gene discovery algorithms confronted with blocks of unknown sequence (‘N-islands’) between likely exons are obliged to interpret the N-island as entirely intronic (since otherwise the algorithm would have to posit splice acceptors, donors, and exonic sequences entirely composed of ‘N’). If this assignment is correct, then the mature mRNA will seamlessly join exon 1 to the left, and exon 2 to the right, of the N-island. Bottom: the N-island could harbor an unknown exon X, incorporated into mRNA between exons 1 and 2. (B) ‘Trinity objects’ are produced by *de novo* assembly of RNAseq reads (Grabherr *et al.* 2011). Top: if such an object is derived from an exact exon 1–exon 2 splice, then it will align in one continuous HSP in BLASTN analysis to the predicted set of mature transcripts. Bottom: in the case where an N-island contains a hidden exon (A, bottom), the Trinity object assembled from the mRNA should have two HSPs separated by Trinity object sequence; the extra sequence is equivalent to exon X.

Blocks of unknown sequence (‘N-islands’, see above) could result from assembly difficulties from blocks of repetitive or low-complexity sequence, which are highly abundant in *Chlamydomonas*; or from regions of DNA that are intrinsically difficult to sequence (*e.g.*, inverted-repeats). Such sequences can cause loss of adjacent high-complexity sequence from the assembly. From the list of Phytozome reference ‘primary transcripts’, we recovered a list of 789 intronic N-islands of varying length. An intronic block of unknown sequence could contain unknown exons (Figure 1A). *De novo* assembly from RNAseq reads could recover the missing high-complexity sequence, even if it is hidden in low-complexity, or difficult-to-sequence, regions in its intronic context.

We carried out a *de novo* transcriptional assembly (see Materials and Methods). The assembled sequences, even after filtering, likely include some assembly artifacts (for example, track-crossing errors at repeat sequences), in addition to authentic *in vivo* mRNA species. We refer to these sequences as ‘Trinity objects’ to reflect initial neutrality concerning their biological status.

### Coverage of splice junctions by Trinity objects

To estimate completeness of the Trinity-generated transcriptome, we performed a BLASTN search with Phytozome transcripts against a database of Trinity-generated objects. Since we wanted to resolve intronic N-islands, we used only Phytozome transcripts predicted to splice out N-islands in the search. There are 821 such transcripts; we analyzed 647 of them, for which we found aligned Trinity objects. These 647 transcripts contain 9720 splice junctions, of which 789 are annotated as splicing out N-islands.

From this set, of 8931 junctions not associated with N-islands, 7141 (80%) were ‘covered’: a Trinity object aligns with continuous coverage across the junction (Figure 1A, top). This result supports the *in vivo* splicing event proposed for these junctions. For 20%, we did not recover Trinity sequences aligned to either side of the splice junction (‘no information’). The ‘no information’ category could reflect many causes, including low mRNA abundance, Trinity alignment difficulties due to repetitive sequence, or incorrect gene models, such as intronic or intergenic sequence predicted to encode a splice junction.

In contrast, of 789 junctions predicted to splice out N-islands, only 272 (34%) were covered (Table S1). There was no information for 396 (50%). For 15%, we found evidence that the N-island-associated junction was associated with hidden exons within the N-island (Figure 1A, bottom, and Table S2).

The striking enrichment of the ‘no information’ category for splice junctions splicing out N-islands suggests that presence of N-islands may correlate with (or cause) difficulties in construction of accurate gene models; this possibility is hard to evaluate directly. We focus below on validating the finding of hidden exons within intronic N-islands. The overall frequency with which intronic N-islands actually contain hidden exons is hard to estimate with confidence due to the large ‘no information’ category; the simplest estimate is 26% (104 with hidden exons out of 393 for which we have Trinity information).

### Hidden exons

If an intronic N-island harbors hidden exonic sequence (Figure 1A, bottom), we may find a Trinity object that ’bridges’ the splice junction using two high-scoring pairs (HSPs) flanking the junction (Figure 1B). Blastn alignments were done with the ’ungapped’ option to force generation of distinct HSPs, rather than introducing gaps in a single HSP. This option was particularly important for discovering very short hidden exons. We also required extra sequence from the Trinity object between the HSPs, to spurious detection of BLAST results with two HSPs that overlap due to direct repeat sequences at the HSP termini. A ’half-bridge’ can result if the Trinity object is too short relative to the amount of missing sequence to make a full bridge (Table S4). To score as a half-bridge, we required that an HSP align within 50 bp of the N island, with a long (> 60% of the Trinity sequence) unaligned tail extending in the direction of the N island. We also required evolutionary support by Blastx alignment of the Trinity sequence to *Volvox* peptides (see below).

### BLAST analysis against Volvox to determine evolutionary conservation of bridging sequences

**Figure 2:**
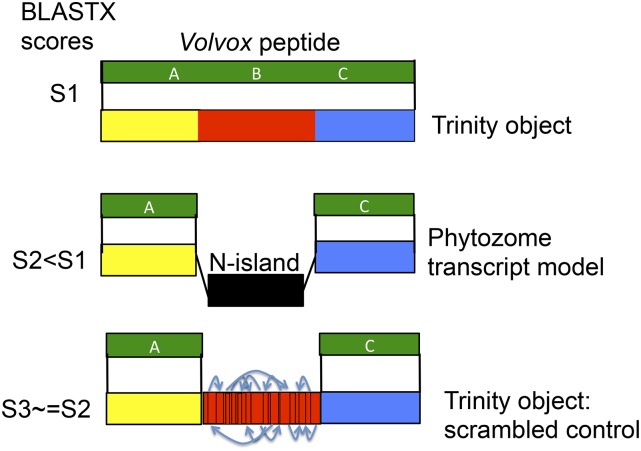
Authentic missing exons can encode evolutionarily conserved sequence: the *Volvox* BLASTX test. Assume that a Trinity object, but not the most closely related Phytozome transcript model, contains a hidden exon (Figure 1). *Volvox* is a close relative to *Chlamydomonas*. If BLASTX of the Trinity object against *Volvox* gives a score, S1, due to alignment to segments A, B, and C, then BLASTX of the corresponding best-hit Phytozome model (A–C) will give a score S2 < S1, due to lacking alignment from the middle segment lost in the N-island. As a control, scrambling the intervening sequence from the Trinity object (random permutation of the amino acid sequence) should eliminate any scoring advantage, yielding score S3 ≈ S2 < S1.

Trinity sequences bridging an N-island-containing splice junction (Figure 1B) suggest identification of missing exons; however, they could result from artifacts of *in silico* assembly. A Trinity object bridging an N-island should frequently improve BLAST alignments to the proteome of *Volvox*, a close relative of *Chlamydomonas*, if the bridge contains genuine translated exons. Such improvement should be highly unlikely if the bridge structure is the result of a Trinity assembly artifact. A similar analysis was useful in evaluating biological significance of annotated 5′-untranslated open reading frames (Cross 2015). Therefore, we carried out BLASTX with nucleotide sequences corresponding to the aligned HSPs plus the intervening sequence provided by Trinity (’extended’) as query, and the *Volvox* proteome as source of subjects. As a control, the intervening sequence was scrambled. We required that queries with and without the intervening sequence both hit the same *Volvox* peptide. To determine how much score increase we should consider significant, we supposed that if the intervening sequence was not translated *in vivo*, then small random score changes should be similar with scrambled controls as with the intact sequence. We found that the scrambled control never showed a score increase > 10, while such increases happened very frequently with the intact sequence. While a BLAST score of 11 is trivially attained in a search of a query against an entire proteome; the reason such a small score is significant in the present case is that the subject for the query is a single *Volvox* peptide preselected on the basis of alignment to the flanking regions common to the reference transcript and the Trinity object (Figure 2). Increases of this magnitude were biologically significant in evaluating upstream open reading frames (Cross 2015). Note that, since the assay is for improvement of BLAST score, the result is specific to the novel segment in question—it is independent of the quality of alignment elsewhere in the sequence. In general, the newly aligned sequence aligns across its length, and lacks stop codons or other indicators of internal errors, though we have not evaluated this comprehensively.

Seventy-seven of 104 sequences that bridge intronic N-islands, and 13 of 13 that half-bridge N-islands, passed this criterion, and thus are highly likely to contain additional exonic sequence. This criterion is conservative, since a substantial proportion of *Chlamydomonas* proteins have either absent or patchy alignment to *Volvox* peptides.

Peptide multiple sequence alignments of translated Trinity objects to Phytozome peptides from *Chlamydomonas* and *Volvox* are found in File S4. Lower-case letters in the Trinity sequence represents novel sequence that was not aligned by Blastn to Phytozome transcripts. The corresponding Trinity nucleotide sequences, with the Blastn-aligned parts in upper case and the unaligned parts in lower-case letters, are given in File S2.

### Alignment of genomic reads to Trinity objects to determine consistency with in vivo RNA splicing

Trinity objects are assembled from short cDNAs. A Trinity object could be: (1) assembled from RNA sequence transcribed from a single contiguous stretch of genomic DNA; (2) assembled from RNA transcribed from discontiguous stretches that are spliced *in vivo* into a single RNA; (3) assembled from RNA transcribed from discontiguous stretches that are combined by Trinity into a single object by misassembly *in silico* (most likely by ‘track-crossing’ at repeat sequences).

**Figure 3:**
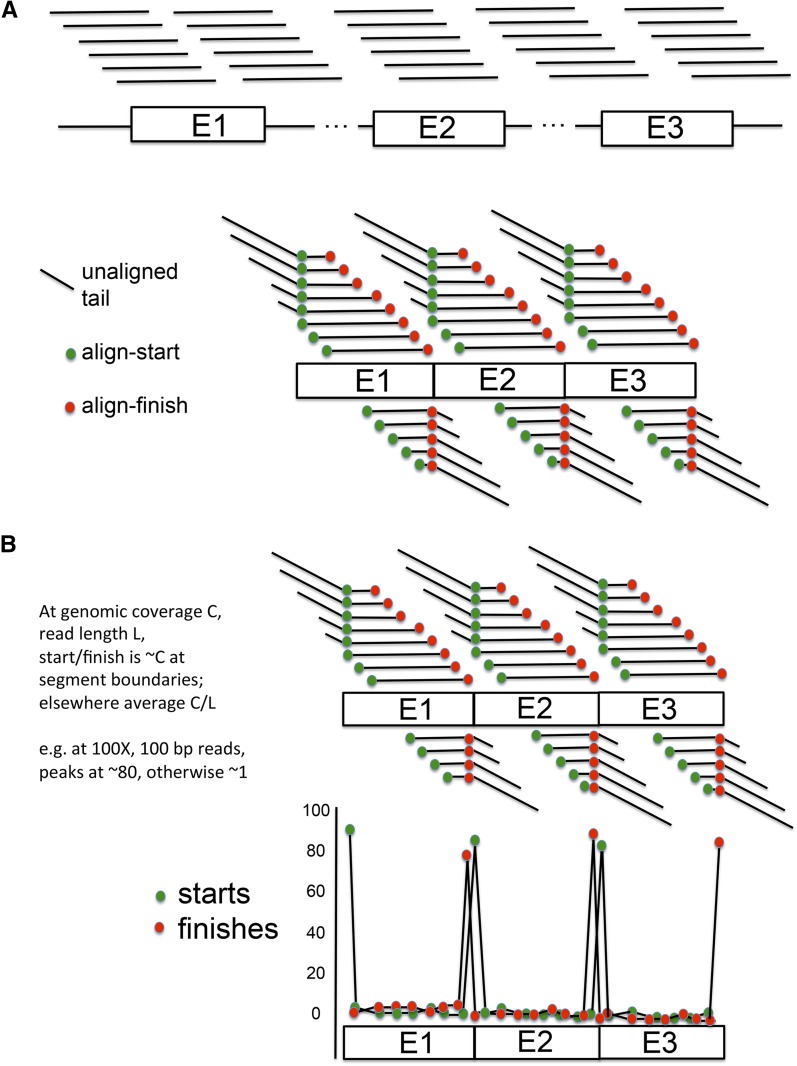
Local alignment of genomic reads to transcripts gives a signature for junctions of discontiguous genomic encoding segments. (A) Tiled Illumina reads derived from the genome covering discontiguous segments E1, E2, and E3 (top) are aligned locally (not requiring alignment across the complete read) to the fused structure E1–E2–E3 (bottom). These reads have starting and finishing positions of alignment to the E1–E2–E3 structure (green and red dots). (B) The result for random starts and finishes of reads in the genome is a huge enrichment of start positions at the beginnings of the genomically discontiguous segments E1, E2, and E3, and for alignment finishes at the ends of E1, E2, and E3 (top). This enrichment is on average the read-length (*e.g.*, 100 × for 100-bp reads). The plot of starts and finishes of alignments by position then yields a highly sensitive indicator of the segment boundaries.

**Figure 4:**
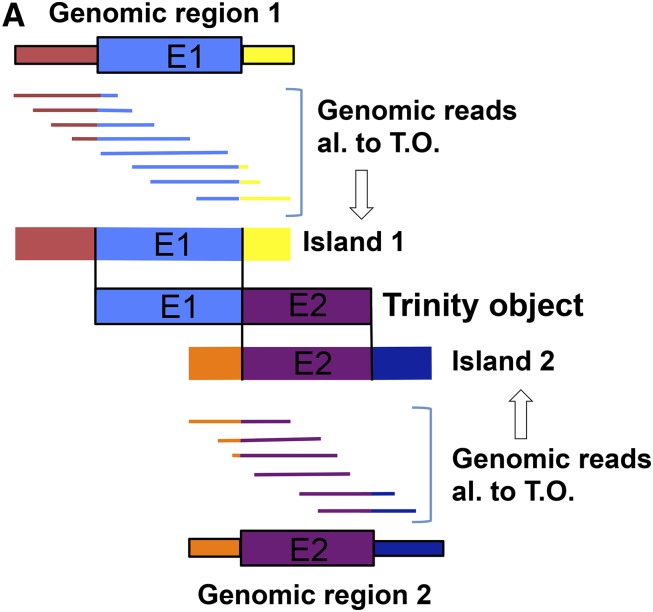

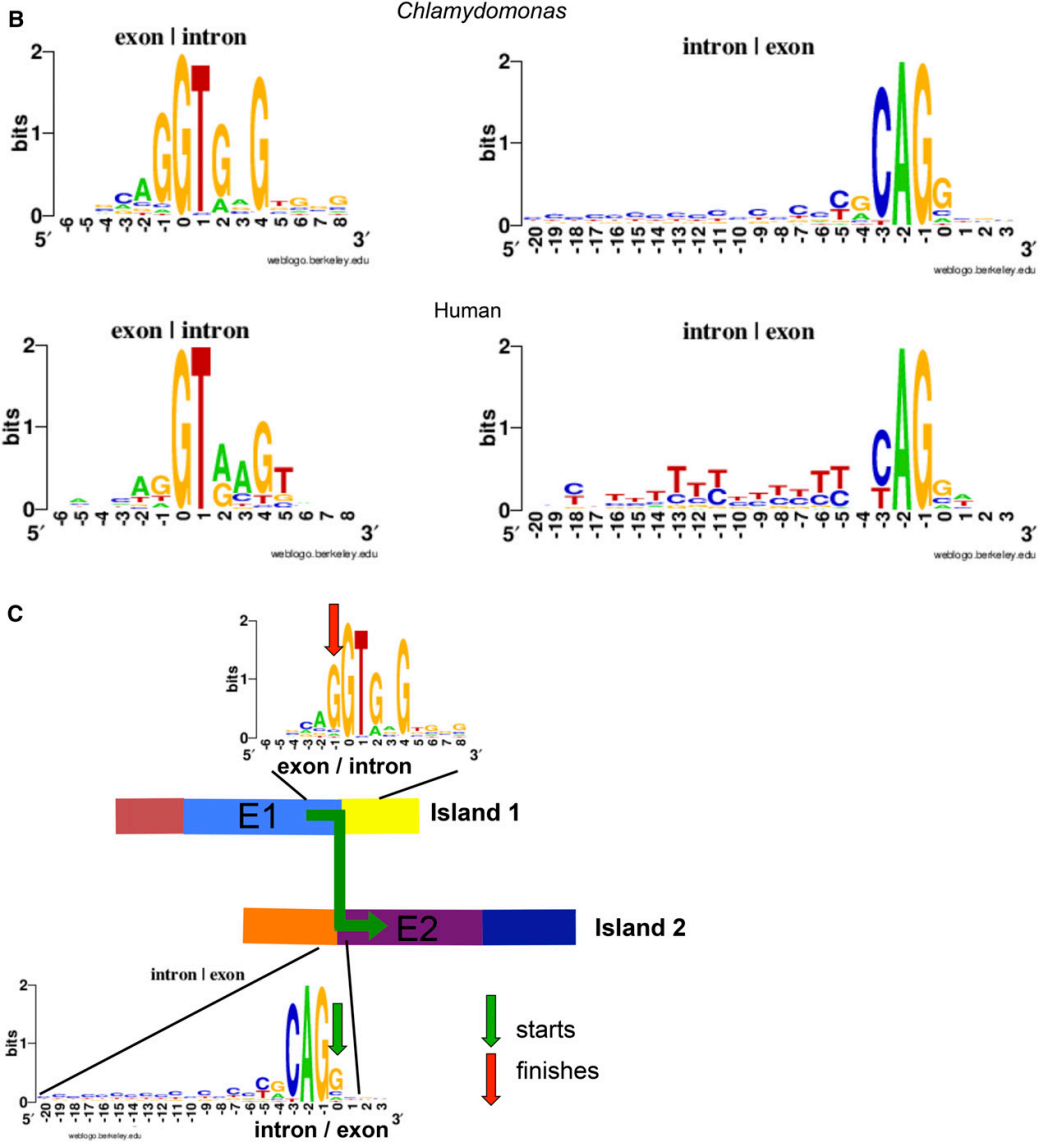
Potential for RNA splicing to assemble consensus islands of genomic sequence into mRNA: the splicing test. (A) Tiled genomic reads (specific segments indicated by different colors) aligned to a Trinity object encoded by two discontiguous segments E1 and E2 yield island consensus sequences (‘Island 1’, ‘Island 2’). Each island has a central segment identical to E1 or E2 and flanking sequences not found in the Trinity object. Note that when aligned within the Trinity ‘frame’, Islands 1 and 2 have no sequence in common, even though each aligns to a large segment of the Trinity object. (B) Consensus sequences for splicing in *Chlamydomonas*. Splice sites from the annotated Phytozome reference genome were collected and analyzed using the Weblogo algorithm (Crooks *et al.* 2004; http://weblogo.berkeley.edu). The human consensus is shown for comparison. (C) The splicing test. Consensus islands assembled as in (B) can be assembled into mRNA only by a join at a highly restricted location [in the absence of any terminal direct repeats, a single nucleotide at the terminus (start or finish) of each island]. This provides a highly constrained condition for searching for splice site homology (donor and acceptor positions; the most likely start and finish positions are indicated by green or red arrows). A series of consensus islands connected by high-confidence splices on the same strand forms a ‘connected island’; a candidate hidden exon should be contained within such a connected island to pass the splicing test.

If a segment of a Trinity object was transcribed from a contiguous stretch of genomic DNA, then genomic reads aligned to the segment should tile over a single continuous matching sequence. In contrast, if the segment was transcribed from discontiguous segments, alignment of genomic reads should break into multiple discrete islands. We aligned 100 bp genomic DNA reads to the collection of Trinity objects with Bowtie2 using ‘local’ alignment (which allows alignment even if only a portion of the read aligns to the template). This leads to a highly specific signature of an alignment discontinuity: an ∼100-fold excess assignment of ‘starts’ and finishes’ of alignment to nucleotide positions exactly at the point where two discontiguous sequences are joined in the template (Figure 3, A and B). These positions were used as seeds to grow a contiguous genomic region out of all reads initially aligned to the Trinity object, by iteratively constructing a majority consensus, discarding reads that fit poorly, and recruiting new reads that aligned well and expanded the consensus. The process terminates when no unrecruited reads both align to the Trinity object and also agree with the growing consensus. The latter criterion is highly effective in discarding reads that align solely by an internal repeat. The result for each discontiguous segment of genomic DNA aligning to part of a Trinity object is an island of sequence supported by a tiled series of reads, with sequence not alignable to Trinity extending from either end (Figure 4A). The extensions are typically of length ∼80 nt, equal to the read length 100, minus the minimum 20 required for Bowtie2 to call a local alignment. Ideally, one such island is found for each discontiguous sequence making up the Trinity object.

Sequential islands of this kind have no aligned sequence at all comparing them to each other, although each has a central segment aligned to sequential segments of the Trinity object. Alignment discontinuities between these islands (the blue-yellow and orange-purple junctions in Figure 4A) mark an obligatory and exact position where the genomic segments must be joined in order to reconstitute the Trinity object. This restricts positions of potential RNA splicing to one nucleotide. (This is expanded to a few nucleotides in the case of direct repeats at the termini of the Trinity-homologous segments). Internal repeats will typically not yield a neighboring start–finish pair in close enough proximity to explain the join by splicing.

Because the position of the join is highly constrained, we reasoned that if the segments were joined by RNA splicing, then we should be able to detect sequence signatures permissive for splicing specifically at these junctions. We examined genomic splice site sequences in the reference assembly/annotation to determine a splicing consensus (Figure 4, B and C). Consistent with previous work in many eukaryotic organisms, the strongest determinant was the /GT…AG/ dinucleotide pair at intron termini; other flanking sequences also contributed significantly to the consensus, equivalent to almost eight nucleotides of specified sequence. We found that a match score of 11 bits (obtained by summing information content for observed nucleotide at each position) was attained by ∼99% of Phytozome annotated splice junctions, and by only ∼1% of randomly selected genomic sequences, and we chose this value as a cutoff for splice junction detection. Because of the near-absolute requirement for the /GT…(intron)…AG/ dinucleotides for splicing, we imposed a two-step test: presence of the dinucleotides, and a score of at least 11 (the dinucleotides alone give a score of 8). We applied this test to the discontinuities in alignment of genomic sequences to Trinity objects, as detailed above.

**Figure 5:**
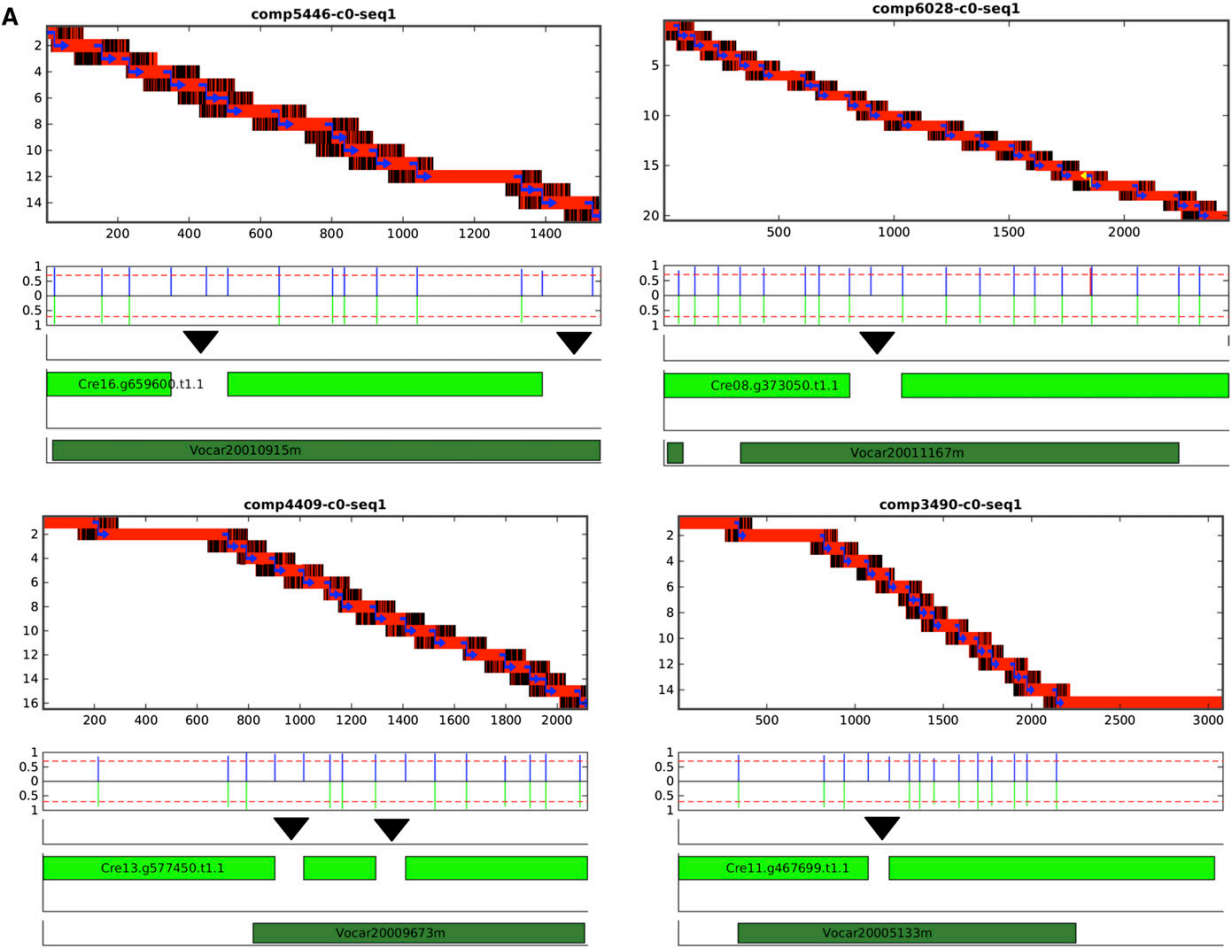

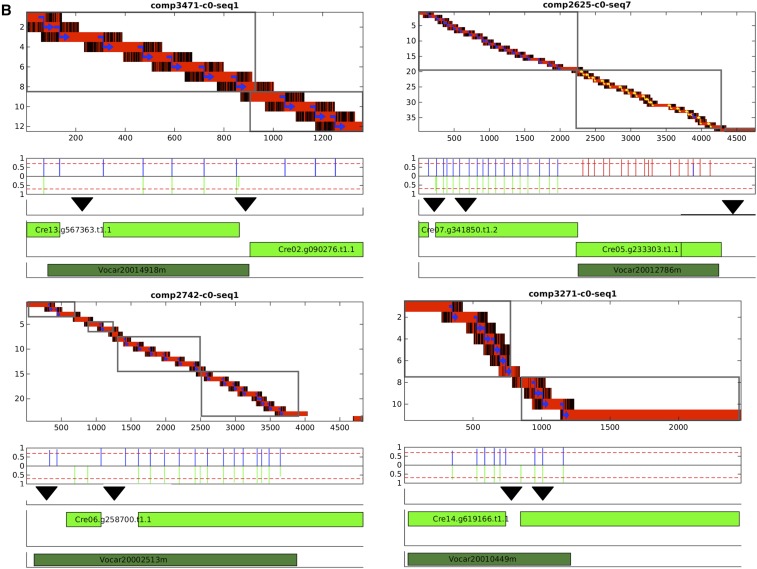
Graphical summary of analysis of Trinity objects. The ‘frame’ for all data are the sequence of the Trinity object (numbers on *x*-axis of top graph). At top is the result of analysis of genomic reads aligned to the Trinity object. (A) The red/black bars are alignment islands, where red indicates identical sequence to Trinity and black non-identical sequence. The blue joining arrows indicate high-scoring splice junctions (if detected, junctions on the opposite strand are in yellow). Below is a quantitative trace of splice junction locations and scores (standardized to the maximum possible score of ∼15; the cutoff value for acceptance of 11 is illustrated by the red line). The blue lines are splices in our model; green lines below are splices from the reference transcript model aligned to the Trinity object. Red lines indicate splice junctions on the opposite strand in the Trinity sequence. The green bars indicate regions of alignment (HSPs) to the specified Phytozome reference transcript. The black triangle indicates that the gap in the Blastn alignment to the Phytozome transcript model is the approximate location of putatively spliced-out N-islands in the reference. The bottom graph indicates the positions of Blastx alignment to the *Volvox* proteome. (A) Typical cases for ‘bridging’ Trinity objects. In these four cases the entire set of consensus islands are joined into one ‘connected island’ by same-sense splices. (B) Rare aberrant cases where the Trinity object is due to ‘track-crossing’ *in silico*, and contains regions of two distinct reference transcripts (top two examples), or where splice detection fails even on the same reference transcript (bottom two examples). In these cases there are multiple connected islands (gray outlines).

The results of this procedure for four typical cases with a ‘bridged’ N-island (Figure 1B, bottom) are shown in Figure 5A. The successive red bars at top represent islands aligned to Trinity; the black lines at left and right of these islands represent non-Trinity-homologous sequence. The blue arrows are high-confidence splice junctions according to the test described above. In the four typical cases in Figure 5A, the algorithm has assembled multiple candidate exons. In all cases. these exons could be joined by high-confidence splices; thus, these sets of sequences form ‘connected islands’ that could be assembled into a single mature transcript by splicing. Additional Matlab-generated diagrams, and the code used for their automated generation, are available in File S1, File S2, File S3, File S4, File S5, File S6, Table S1, Table S2, Table S3, Table S4, and Table S5.

Also indicated are positions of splice sites in the aligned reference annotation compared to those detected in the island termini. The splice junctions are almost always are on the same strand; one case where a splice junction could be called on both strands can be noted in Figure 5A, top right (yellow arrow).

The exact lineup of almost all computed and reference splice junctions constitutes strong evidence for accuracy of our procedure, and supports these intron locations in the reference annotation. The exceptions to this rule are essentially all detected at the position of N-islands thought to be spliced out according to the reference, that are in ∼25% of cases associated with additional exons in this analysis. Importantly, almost all of the nucleotides contributing to the splice site consensus are intronic (Figure 4B), and thus not present in the Trinity-assembled sequence. Finding these consensus sequences in the genomic tails at the junction is thus entirely independent validation.

Examples of the main types of occasional aberrant patterns are shown in Figure 5B. The boxes delineate ‘connected islands’ defined by splice junctions on the same strand. In the top left and top right of the figure, Trinity assembly errors in which two transcripts were fused computationally into single Trinity objects are evident. Note that, in the case at top right, the fusion switched the strand of the sequence, as indicated by splice junction orientation (blue to yellow). The bottom two cases are more likely to be simple failure of detection of splice junctions by the algorithm.

### Case studies: PF20 and CDC27

We will discuss two cases of exon discovery in detail. *PF20* is required for flagellar function; its complete cDNA sequence encoding 606 amino acids was described previously (Smith and Lefebvre 1997), but an intronic N-island in the annotated reference genome (between exon 8 and 9 in the Phytozome gene model Cre04.g227900) eliminated coding sequence for amino acids 357–488.

**Figure 6:**
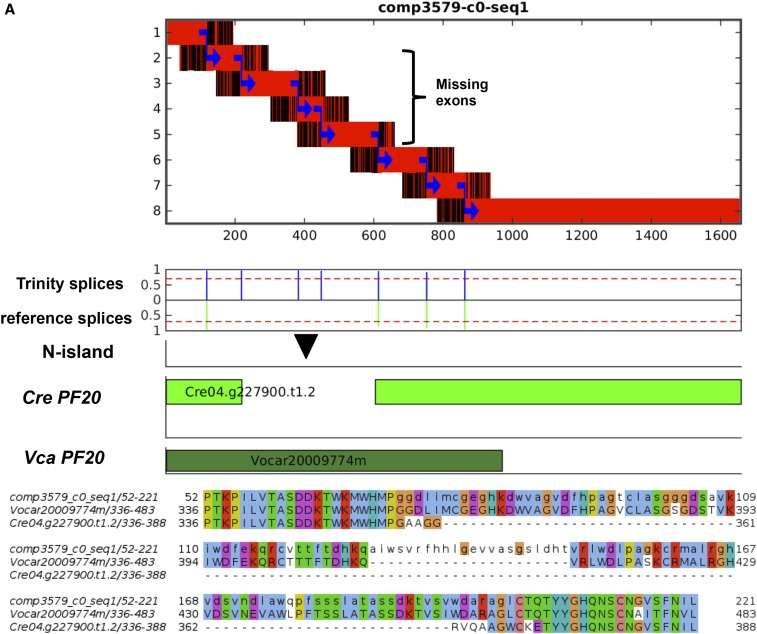

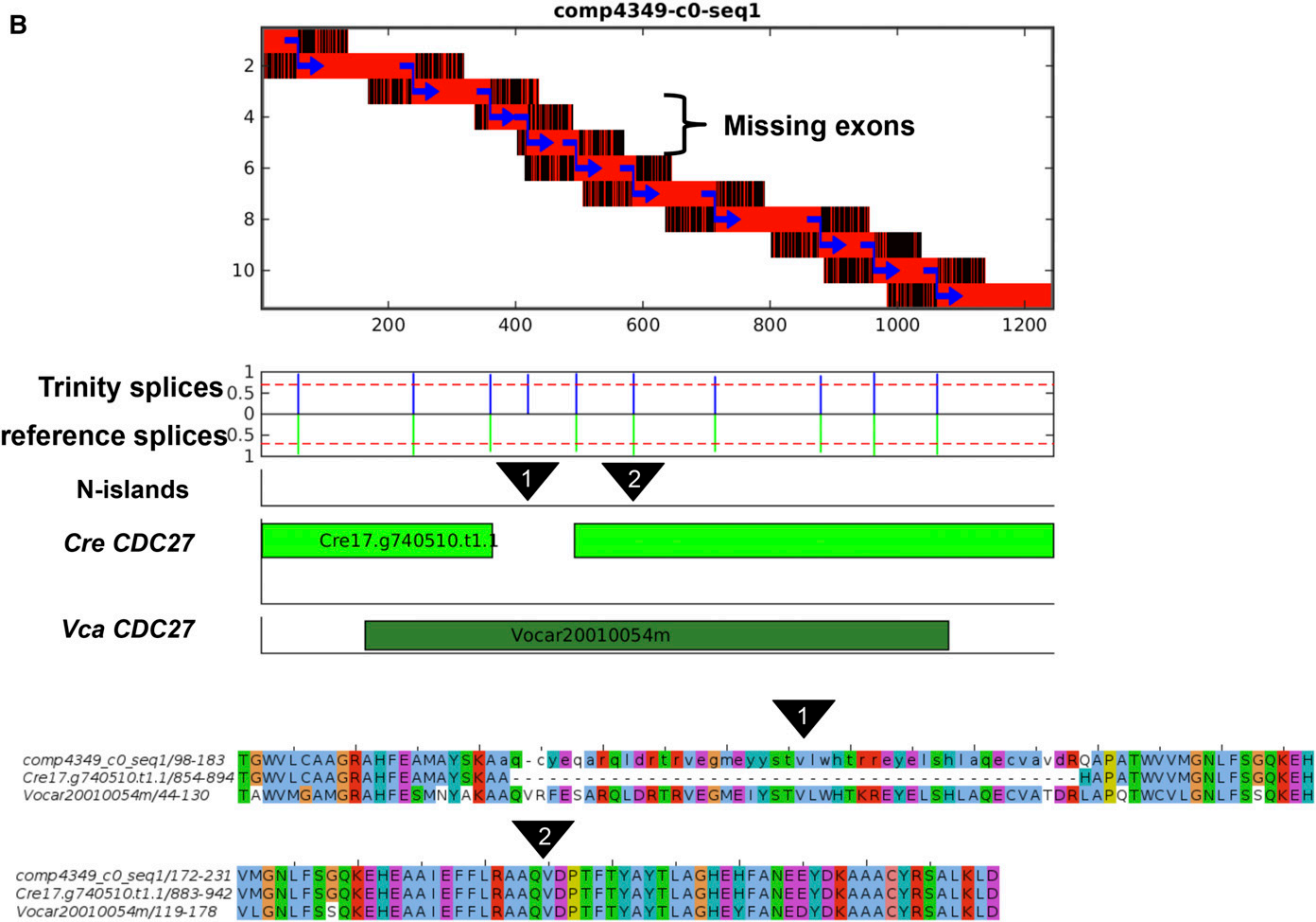

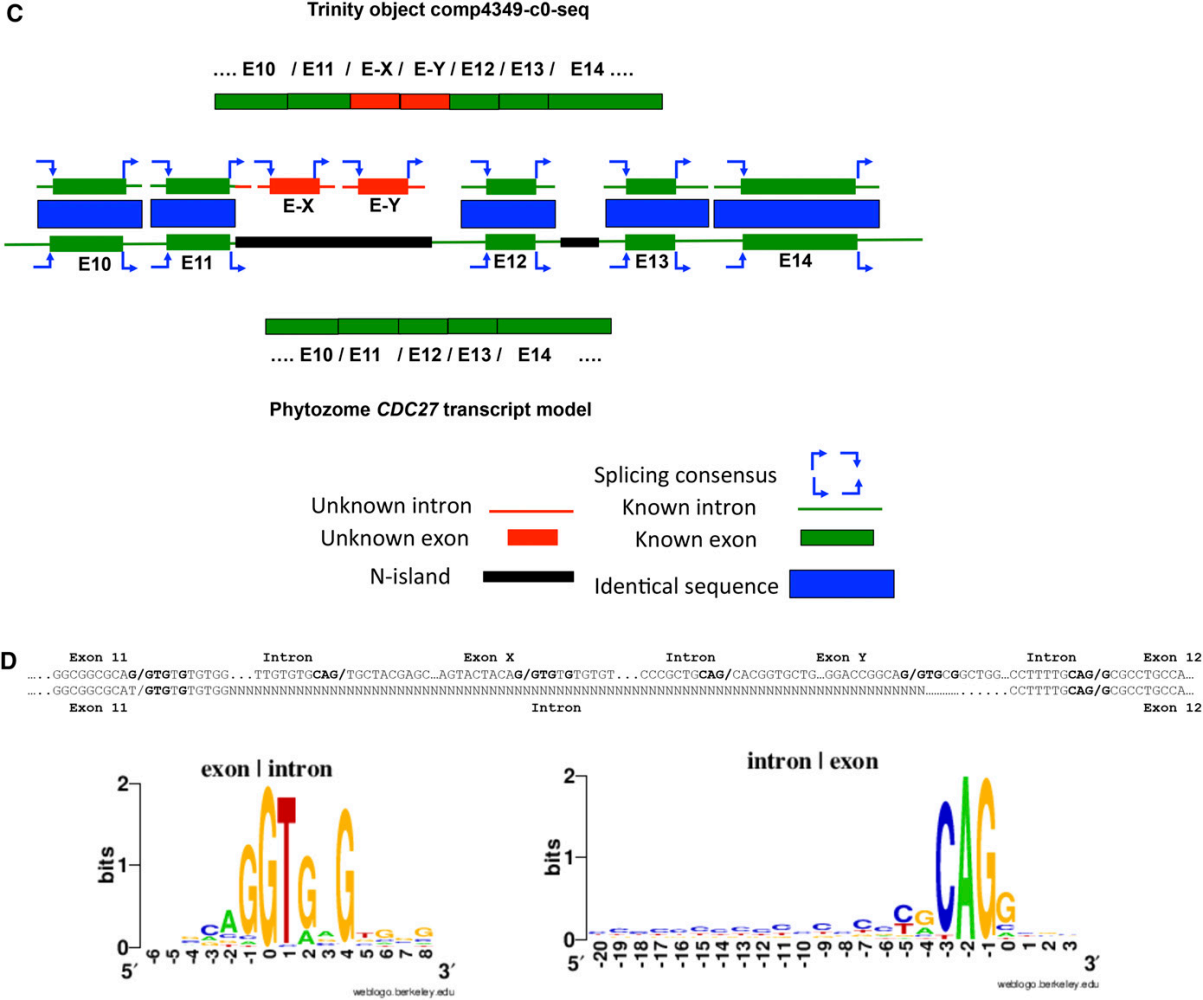
N-islands can contain hidden exons. Two examples are shown. (A) *PF20*; (B, C) *CDC27*. (A, B) Diagrams as in Figure 5. In both cases, one N-island contains hidden exons (four in *PF20*, two in *CDC27*). The second N-island in *CDC27* is covered; the diagram shows it to be authentically intronic according to the Trinity object analysis. As in Figure 5, black bars indicate the approximate position of the intronic N-island in the Phytozome gene model. Multiple sequence alignments between Trinity and Phytozome peptide sequences from *Chlamydomonas* and *Volvox* were generated by clustalo (Sievers *et al.* 2011). (C) The analysis recovers splice-site-proximal intronic sequences; illustrated for *CDC27*. The islands from genomic reads, including Trinity nonhomologous sequence, align perfectly with reference genomic sequence for individual exons (green bars), and surrounding intronic sequence (green lines). Similar extensions (red lines) beyond the Trinity-aligned segments (red bars) are detected for the two hidden exons ‘E-X’ and ‘E-Y’. (D) These extensions are characterized by strong terminal splice site homology, which we take as evidence that ‘exons X and Y’ are in fact spliced into the mature *CDC27* mRNA, as reflected in the Trinity object comp4349-c0-seq1.

Figure 6A diagrams our results for *PF20*, which replace the N-island with four exons. Inclusion of these exons in the predicted mRNA results in a complete coding sequence exactly matching the results of Smith and Lefebvre (1997). The bottom dark green bar in Figure 6A indicates regions of homology to *Volvox* PF20. The bridging sequence is required for optimal alignment to *Volvox*. This result, recapitulating a careful single-gene study, was generated by unsupervised computations, with the only input being Illumina libraries from bulk cDNA and genomic DNA.

CDC27 (*Chlamydomonas* Cre17.g740510) is a conserved component of the anaphase-promoting complex (Zachariae and Nasmyth 1999). The Cre17.g740510 gene model has two intronic N-islands, between exons 11 and 12 (246 N) and between exons 12 and 13 (100 N). We identified a Trinity object that covered exons 9–17 of the *CDC27* transcript model (Figure 6B). Two separated HSPs surround the first N-island, with extra sequence between the HSPs in the Trinity object [the signature for possible hidden exons (Figure 1)]. The first HSP aligns to reference exons 9–11, and the second HSP covers reference exons 12–17, including the 3′UTR. The intervening “missing sequence” between exons 11 and 12 is accounted for by splicing in two new exons ‘X’ and ‘Y’ from a previously unknown genomic segment; the extra peptide sequence thereby added to CDC27 is supported by *Volvox* homology (Figure 6B). Indeed, this extra peptide sequence is conserved in CDC27 from a wide range of eukaryotic species including *Arabidopsis*, animals, and yeast. In contrast, the second annotated intronic N-island (triangle in Figure 6B) is ‘covered’, in agreement that this N-island is completely contained within an intron (Figure 6B).

The Trinity-nonhomologous ‘tails’ at 5′ and 3′ ends of the aligned islands are likely to be intronic. Indeed, these sequences for ‘covered’ introns exactly matched reference splice donor and acceptor, and neighboring intronic sequences, at each covered splice junction (Figure 6D); thus, splice junction-proximal intronic sequences are accurately recovered by local alignment of genomic reads to Trinity objects, independent of any prior reference or annotation.

### Exonic N-islands

Most N-islands are annotated as intronic. There are, however, 40 exonic N-islands in the reference assembly. Most are assigned to 3′UTR or 5′UTR. We found three cases where a Trinity object bridged an exonic N-island (Table S3). In two of these the bridge resulted in a significant coding sequence improvement in Blastx score against *Volvox*.

### Flanking N-islands

**Figure 7:**
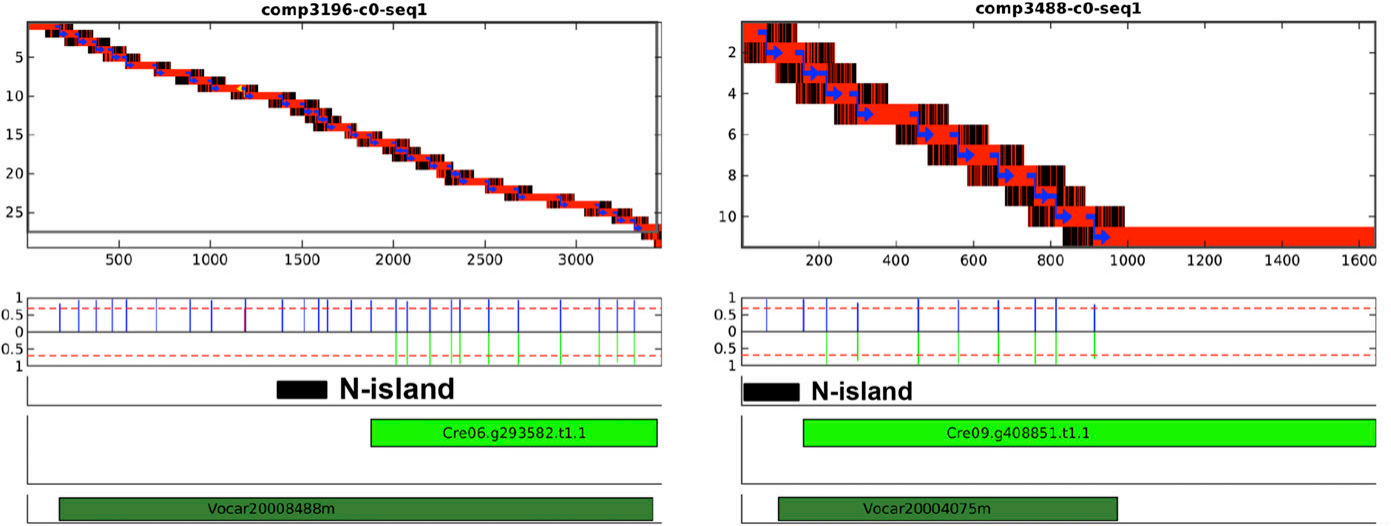
Flanking N islands can truncate a gene model. Diagrams as in Figure 5. Two Phytozome transcript models with flanking N islands are shown, Cre06.g293582.t1.1 and Cre09.g408851.t1.1 (light green bars). The Trinity objects comp2196_c0_seq1 and comp3488_c0_seq1, respectively, align to the body to the Phytozome transcripts, and extend into the N islands (represented by black bars). The Trinity sequence in the extensions (unaligned to *Chlamydomonas* sequence) can be aligned by Blastx to *Volvox* proteins (dark green bars).

The intronic and exonic N-islands examined above were all annotated within the boundaries of a gene. Some N-islands flank an annotated gene; such a gene might actually extend into (or even beyond) the flanking N-island. We defined a flanking N-island as an N-island starting within 100 bp of the 5′ or 3′ borders of a gene model. If the authentic gene extends into the N-island, then there could be a ‘half-bridging’ Trinity object aligning at one end to the reference gene model, with the other end ‘hanging’ in the N-island. We scored 136 flanking N-islands in the genome, 11 of which could be composed as a connected island extending into the N-island, significantly increasing the *Volvox* BLAST score (Table S5). Two examples are presented in Figure 7. Because the Trinity objects are short, it is unlikely that these extensions complete the transcript model, and the lack of a full bridge intrinsically makes these hits less reliable, but it is clear that extension of these methods could significantly extend or even complete gene models by foraging into the adjacent N-islands. We suspect that the low yield of these structures is related to the sequencing difficulties (simple sequence, repeat regions, and inverted repeats) that led to them being called as N-islands in the original annotation.

## Discussion

N-islands are regions of undetermined sequence whose lengths are only estimated. Many N-islands are assigned in the reference to intergenic or intronic regions, and such assignments are problematic (since, for example, intronic N-islands could, in principle, contain any number of unknown exons, or even complete unknown genes). Our results suggest additional internal coding exons, or N- or C-terminal extension of coding sequence, to replace the putatively noncoding N-islands, in a significant minority of N-islands.

We did not attempt to validate the new exons by additional direct experimentation; possibilities would include longer RNAseq reads, paired-end, or strand-specific RNAseq, and different sequencing technologies allowing much longer genomic DNA reads. However, the BLASTX improvement assay, and the finding that high-confidence splice junctions could be formed at almost all the relevant junctions, constitute two entirely independent ways to validate the results, since neither of these results is predicted if the new candidate exons are not spliced and translated as predicted.

The methods used here are useful for ‘proofreading’ a reference assembly/annotations, but the methods do not require a prior reference. In principle, these methods could validate RNAseq-based transcriptome assemblies, including providing genomic splice sites and flanking intronic sequences, even for organisms without any assembled genome or annotation.

While our computations *per se* did not use the assembled genome or reference annotation, the results could be evaluated with much higher confidence when looking for a revision of the current assembly, rather than entirely *de novo* sequence. Thus, we have more confidence in the bridged than the half-bridged cases (such as with the flanking N-islands), and we did not so far attempt to evaluate Trinity objects that were completely unalignable to any reference transcript. One limitation to our approach, as noted above, was the use of single-end, nonstrand-specific 50-nt RNAseq reads. More informative libraries might improve resolution and reduce track-crossing sufficiently that a reliable full transcriptome, along with junctional sequences at promoter, termination and splice sites, might be recovered *de novo*. The *Chlamydomonas* genome is a favorable case in that it is 1.2 × 10^8^ bp, of which around one-third is exonic. It is unfavorable in having a very high density of repeats and low-complexity sequence (likely the source of most alignment difficulties including N-islands, and certainly the source of most difficulties we encountered with our approach).

In principle, the genomic islands recovered to account for Trinity objects could be used iteratively to align genomic reads, ultimately finding the next exon and providing sequence of the entire intron. We have not attempted this because of the clear danger of track-crossing in repeat sequences, which are especially enriched in intronic sequence, but it is likely possible in at least some cases.

The test for splice sites at the borders of the Trinity-nucleated genomic islands also tests the validity of the Trinity object, since it is a sensitive indicator of track-crossing leading to fusion transcripts (Figure 5B). This could be a useful proofreading step when Trinity-generated transcriptomes are used in the absence of a reference genome sequence, since it requires only a single library of genomic reads aligned to the Trinity objects, and requires no prior assembly of a reference genome.

The combination of *de novo* transcriptome assembly followed by alignment of short genomic reads onto the assembled transcriptome objects in principle represents a massively parallel approach to validating a genome assembly/annotation, equivalent in some ways to checking a near-complete collection of ESTs against the annotation in a single experiment. Our implementation was not quite up to this challenge, because the assembly was only ∼70% complete, and a significant minority of the assembled objects had track-crossing and other issues (Figure 5B). However, these seem likely to be solvable issues, through the use of better RNAseq libraries, and through making use of paired-end information for both RNAseq and DNA libraries.

While our approach did uncover some novel exons missed in the prior assembly/annotation, overall our results document the very high quality of the annotation. A majority of N-islands annotated as intronic most likely are purely intronic, and a large majority of annotated splice junctions lacking N-islands are confirmed: they are covered by continuous Trinity object sequence, and put together from discontiguous genomic segments with high-confidence splices.

The current assembly/annotation reports ∼4 Mb of Ns (3.4% of the genome), distributed in 1489 islands of highly variable size; 64% of these islands are located in introns, and these are primarily the N-islands addressed by the current study. However, these intronic N-islands are short (only 15% > 1 kb, 2% > 10 kb), and account for only 22% of the total Ns; most Ns are found in large islands (45% > 1 kb, 25% > 10 kb) annotated as intergenic. It is likely that at least some of these large regions contain unknown transcripts, which should be represented in our study by Trinity objects that did not align to any current Phytozome transcript model. We have not examined this question in detail, but it is clear that few such objects exist in our dataset, and could account for no more than a very small proportion of the 3.1 Mb of Ns in intergenic islands. Therefore, while we cannot make a strong conclusion on this point, it seems at least plausible that most of the large intergenic N-islands are composed of noncoding sequence; perhaps sequence characteristics leading experimentally to N-island status are relatively rare in sequences transcribed into mRNA. Until the sequences are identified, this remains a speculation.

## Supplementary Material

Supplemental Material
