# Supplementary material for: Patching Holes in the Chlamydomonas Genome
Source: G3 (Bethesda). 2016 May 10;6(7):1899–910. doi: 10.1534/g3.116.029207 (PMC4938644; doi:10.1534/g3.116.029207)
Supplement: Supplemental Material [file supp_6_7_1899__index.html]

Patching Holes in the Chlamydomonas Genome — Patching Holes in the Chlamydomonas Genome — Supplemental Material 

# Patching Holes in the *Chlamydomonas* Genome

## Supplemental Material for Tulin and Cross, 2016

**Files in this Data Supplement:**

- File S1 - Supplemental information provided with manuscript. (.pdf, 105 KB)
- File S2 - Supplemental Fasta. (.zip, 3 MB)
- File S3 - Supplemental Perl scripts. (.zip, 77 KB)
- File S4 - Supplemental alignments. (.zip, 256 KB)
- File S5 - Supplemental Matlab. (.zip, 6 MB)
- File S6 - Matlab sample analysis. (.zip, 5 MB)
- Table S1 - Phytozome N-island-containing transcripts where the assigned intronic location of the N-island is consistent with our analysis. (.xlsx, 19 KB)
- Table S2 - Phytozome N-island-containing transcripts, for which we identified a Trinity object that 'bridges' an intronic N-island, indicating that the N-island contains hidden coding sequence. (.xlsx, 13 KB)
- Table S3 - Phytozome N-island-containing transcripts, for which we identified a Trinity object that bridges an exonic N-island, thus 'filling in' the missing sequence. (.xlsx, 7 KB)
- Table S4 - Phytozome N-island-containing transcripts, for which we identified a Trinity object that 'half- bridges' an intronic N-island, suggesting that the N-island contains hidden coding sequence. (.xlsx, 8 KB)
- Table S5 - Phytozome transcripts with an N-island located 5' or 3' to the gene body. We identified Trinity objects that 'half-bridged' these N-islands. (.xlsx, 8 KB)
