# Supplementary material for: Patching Holes in the Chlamydomonas Genome
Source: G3 (Bethesda). 2016 May 10;6(7):1899–910. doi: 10.1534/g3.116.029207 (PMC4938644; doi:10.1534/g3.116.029207)
Supplement: Supplemental Material [file supp_g3.116.029207_FileS1.pdf]

Supplemental Information provided with manuscript

SUPPLEMENTAL_TABLES
These tables list N-island-containing Phytozome transcripts.
Supplemental_Table_1_COVERED
Phytozome N-island-containing transcripts where the assigned intronic location of the N-island is consistent with our analysis.
Supplemental_Table_2_intronic_BRIDGED
Phytozome N-island-containing transcripts, for which we identified a Trinity object that 'bridges' an intronic N-island, indicating that the N-island contains hidden coding sequence.
Supplemental_Table_3_exonic_BRIDGED
Phytozome N-island-containing transcripts, for which we identified a Trinity object that bridges an exonic N-island, thus 'filling in' the missing sequence.
Supplemental_Table_4_intronic_HALFBRIDGED
Phytozome N-island-containing transcripts, for which we identified a Trinity object that 'half-bridges' an intronic N-island, suggesting that the N-island contains hidden coding sequence.
Supplemental_Table_5_FLANKING
Phytozome transcripts with an N-island located 5' or 3' to the gene body. We identified Trinity objects that 'half-bridged' these N-islands.

SUPPLEMENTAL_FASTA (File S2)
Supplemental_Fasta_1_all_Trinity_assembled_sequences.fa
Fasta file containing the 3114 raw Trinity-assembled sequences (Trinity objects) that we analyzed.

In Supplemental_Fasta 2-5, uppercase letters indicate sequence that was aligned by Blastn to existing Phytozome transcript models. Lowercase letters represents unaligned sequence (i.e. sequence present in the Trinity object, but potentially missing from the genome assembly).

Supplemental_Fasta_2_intronic_bridged.fa
Supplemental_Fasta_3_exonic_bridged.fa
Supplemental_Fasta_4_halfbridged.fa
Supplemental_Fasta_5_flanking.fa

SUPPLEMENTAL_PERL_SCRIPTS (File S3)
Perl script myNisland_accounting.pl used to run the analysis.

SUPPLEMENTAL_ALIGNMENTS (File S4)
Peptide alignments between Trinity objects (File S2) and Phytozome sequences (*Chlamydomonas* and *Volvox*).
Alignments were done by Clustalo using default parameters. To aid visualization, the alignments were sliced to focus on the segments containing new sequence (corresponding to lowercase letters in File

S2).
Supplemental_Alignments_1_COVERED.txt
Supplemental_Alignments_2_intronic_BRIDGED.txt
Supplemental_Alignments_3_exonic_BRIDGED.txt
Supplemental_Alignments_4_intronic_HALFBRIDGED.txt
Supplemental_Alignments_5_FLANKING.txt

SUPPLEMENTAL_MATLAB (File S5)
Matlab script used in the analysis.

MATLAB SAMPLE ANALYSIS (File S6)
A subset of the raw data is provided in the file 'Matlab_sample_analysis_and_README.zip'. This raw data can be analyzed with the Matlab script (File S5).

Overview of computational pipeline

Generating the Trinity assembly and alignment of genomic reads
Single-end 50 bp reads from RNAseq time-courses (Tulin and Cross, 2015) from six libraries were used. We collected all reads that mapped to gene models that contain internal (intronic or exonic) and flanking N-islands (Trinity6_Nisland_genes.bed). We also included unmapped reads from two libraries. In total, 57 million reads were used as input to Trinity (Haas et al. 2013).

Trinity was executed as

```
$ Trinity.pl --seqType fq --JM 70G --single ../../Trinity_input_data/Trinity6_input_data/Trinity6_catenated_input.fq --
output .
```

Trinity generated 10134 sequences. These were filtered down to 3473 sequences by requiring a maximal e-value of 0.01 to either Arabidopsis TAIR10, or Volvox 2.0 peptides. To reduce redundancy within this set, we filtered again with the usearch program (http://www.drive5.com/usearch/):

```
$usearch -cluster_fast Trinity6_blastx_filtered.fa -sort length -id 0.8 -strand both -centroids
```

This gave the final set of 3114 Trinity objects that we analyze in the paper. To facilitate downstream analysis, we took the reverse complement of Trinity objects, where necessary, to match the strand of the top Blastn hit to Phytozome *Chlamydomonas* primary transcripts.

A bowtie2 index was generated from the 3114 Trinity objects, and genomic 100 bp single-end reads from wild type Chlamydomonas (CC-124) were aligned by bowtie2:

```
$perl /usr/local/bin/bowtie2   --local -p 3 --no-unal --mp 20 --rdg 20, 20 -x ${INDEX_GENOME} -U \
${SOURCE_DIR}/catenated_fastq_files.fq.gz | samtools view -bS - > ${TAG}.aligned.bam 2> ${TAG}_stderr.txt
```

The genomic reads used corresponded to ~100x coverage of the *Chlamydomas* genome.

The mismatch (--mp) and read-gap (--rdg) penalties were set higher than the defaults (--mp 6; --rdg 5,3) to promote perfect alignments to the Trinity sequence. We expected no polymorphisms, since the Trinity assembly and the genomic reads come from the same strain background.

The alignment to Trinity sequences was used to build 'connected islands' by Matlab (Figure 5, 6)

Analysis of Blastn alignment between Trinity and Phytozome transcript models

We performed a Blastn alignment between the strand-corrected Trinity objects and Phytozome primary transcript models:

```
$ blastn -query accessory_files/Creinhardtii_281_v5.5.transcript_primaryTranscriptOnly.fa -db ~/blast_db/Trinity6_strand_corrected_raw -ungapped -out Creinhardtii_281_v5.5.transcript_primaryTranscriptOnly_vs_Trinity6_strand_corrected_raw_blastn_ungapped.bls
```

The Blastn alignment was analyzed by 'myNisland_accounting.pl' and accessory files in Supplemental Files according to the basic algorithm:

1. Read next Blastn result.
2. Check if the query (Phytozome Primary transcript) has flanking N islands.
    If yes, score the flanking N island, then proceed to 3.
    If no, proceed to 3.
3. Check if the query contains intronic or exonic N islands.
    If NO, return to 1.
    If YES, for each N island, do the following.
    <u>IF ONLY 1 HSP</u>
        Check if the N island is COVERED.
        If yes, continue to next N island in current Phytozome transcript OR if there are no more N islands, return to 1.
        If no, check if the N island is HALF-BRIDGED. Then continue to next N island.
    <u>IF >1 HSP</u>
        Check if the N island is COVERED.
        If yes, continue to next N island OR if there are no more, return to 1.
        If no, check if the N island is BRIDGED. Then continue to next N island.
4. If there are no more N islands in the current Phytozome model, return to 1.

COVERED: An intronic N island is marked by two adjacent nucleotides in the Phytozome transcript sequence. If there is a Blastn HSP to a Trinity object that extends across the N-island-junction, we considered this evidence for the Phytozome assignment of the N-island as fully intronic. We required that the Trinity object be contained within a 'connected island' that extends at least 100 bp to the left and right of the N-island-junction.
A covered N-island-junction is shown in Figure 1B (top panel).

BRIDGED: If a single Trinity object aligns with one HSP on either side of the N-island-junction, with unaligned Trinity sequence between the two HSPs, we called the N island BRIDGED. To score as BRIDGED, we also required that

1. the Trinity object be contained within a 'connected island' that extends at least 100 bp to the left and right of the unaligned Trinity segment.
2. <100% of the Trinity object is aligned

A bridged N-island-junction is shown in Figure 1B (bottom panel).

HALF-BRIDGED: We considered the possibility that the Trinity sequence may in some cases be too short to complete a full bridge. In such cases, the Trinity object would align (Blastn) with a single HSP on either the left, or the right side of the N-island-junction, with an unaligned 'tail' extending in the direction of the N island.

To score a half-bridged intronic N island, we required:

1. that the HSP ends (if on the left side) or begins (if on the right side) within 50 bp of the N-island-junction.
2. that the HSP does not overlap by more than 10 bp from the left to the right side or vice versa.

10 bp is accepted since this occurs frequently by Blastn alignment due short sequence repeats at the splice junction.
3. that the HSP length (aligned sequence) is <40% of the Trinity length. This is a subjective cut-off to focus on cases where a substantial proportion of the Trinity sequence is unaccounted for.
4. that a 'connected island' span at least 200 bp around the border between the HSP and the unaligned tail.

In addition, due to the lower confidence in half-bridges (since they are not anchored on both sides of the N-island-junction), we required a sequence-dependent score increase of 10 or more by the Blastx test to Volvox described in the main text, i.e. the half-bridges require support from evolutionary conservation.

FLANKING: To identify cases where an N island may cause premature termination of a gene model, we looked for Trinity objects that align (Blastn) within the transcript body, with an unaligned 'tail' extending in the direction of a flanking N-island (N-island assigned to lie outside the 3' of 5' borders of the gene). As for the half-bridged N-islands, we required support from a 'connected island' at the HSP-tail border, and evolutionary support from the Blastx test to *Volvox*.

Result

A total of 40 exonic, 136 flanking and 789 intronic N islands were analyzed. This is not the full set of N islands in the *Chlamydomonas* genome (v5.3.1); we limited the analysis to those N-island-containing transcripts that were the top hit of some Trinity object by Blastn. This was done to prevent scoring N islands based on spurious Trinity-Phytozome alignments.

We scored

272	COVERED N islands	Supplemental Table 1
104	intronic BRIDGED N islands	Supplemental Table 2
4	exonic BRIDGED N islands	Supplemental Table 3
13	intronic HALF-BRIDGED N islands	Supplemental Table 4
11	FLANKING N islands	Supplemental Table 5

Alignments

We constructed nucleotide sequence alignments (clustalo, Sievers et al. 2011) between the Trinity objects and their corresponding Phytozome transcript models (Supplemental Alignments). The files are labeled as:
Cre01.g003532.t1.1_comp5218_c0_seq1.aln
where Cre01.g003532.t1.1 is the Phytozome identifier of the N-island-containing transcript, and comp5218_c0_seq1 is the Trinity object containing new sequence information.

We constructed peptide multiple sequence alignments between Trinity, *Chlamydomonas* and *Volvox*. These alignments were 'sliced' to focus on the new sequence information provided by Trinity. In general, the Trinity translations show an unbroken ORF across the unaligned (new) sequence. Moreover, while unalignable to *Chlamydomonas*, this sequence frequently aligns to *Volvox*. This sequence-dependent score improvement was quantified by the Blastx test described in the main text ('BLASTX test' in Supplemental Tables 2-5).
Peptide alignments are labeled as:
Cre01.g003532.t1.1_comp5218_c0_seq1_sliced.aln.

In all alignments, lower case letters in the Trinity sequences represent the candidate new exonic sequence.

How many N islands are likely correctly assigned to introns?
Global coverage rate for splice junctions is ~70%, meaning 30% lack information due to the incompleteness of the Trinity assembly. The part (~789/10000 = 7.89%) that is relevant to bridged N islands is ignored here.

There are 789 N-island-junctions with an intronic N-island. If all of these are correct, we expect to see 70% covered, 30% no information:
552 covered
236 no info

We observe
272 covered (34%)
108 bridged (14%)
13 half-bridged (1.5%)
396 no info (50%)

This is roughly half the number of covered junctions we would expect if all were correct.
The proportion of no information junction is also higher that the expectation.

The lower bound for correct N islands is: 272/789 = 34%
In the sample of 789 junctions we expect 236 no information junctions. If the remaining 160 are correct, we have 54% correct N-islands.

References

Haas, B.J. et al. (2013). De novo transcript sequence reconstruction from RNA-seq using the Trinity platform for reference generation and analysis. Nat. Protoc. **8**: 1494–512.

Sievers, F., Wilm, A., Dineen, D., Gibson, T.J., Karplus, K., Li, W., Lopez, R., McWilliam, H., Remmert, M., Söding, J., Thompson, J.D., and Higgins, D.G. (2011). Fast, scalable generation of high-quality protein multiple sequence alignments using Clustal Omega. Mol. Syst. Biol. **7**: 539.
