# Supplementary material for: Patching Holes in the Chlamydomonas Genome
Source: G3 (Bethesda). 2016 May 10;6(7):1899–910. doi: 10.1534/g3.116.029207 (PMC4938644; doi:10.1534/g3.116.029207)
Supplement: Supplemental Material [file supp_g3.116.029207_FileS5.zip › File_S5_Matlab/Trinity_Matlab_readme.docx]

**Information about Matlab scripts used to analyse alignment of genomic reads to a Trinity-assembled ‘transcriptome’**

From within the folder ‘trinity_genomic_alignment, run

tga_structure = setup_trinity_objects('Trinity6_strand_corrected_raw.fa', 'query_anchored_coords_20151019.txt')

to generate a structure that contains information about each Trinity object. The function ‘setup_trinity_objects’ requires:

1. Trinity6_strand_corrected_raw.fa: fasta file with Trinity objects to be analysed

2. query_anchored_coords_20151019.txt: information about Blastn and Blastx alignment of Trinity objects to Chlamydomonas and Volvox

3. sam files from alignment of genomic reads to Trinity objects (one file per Trinity object). These are in the subdirectory ‘trinity_data_files_G3_submission/’. The file needs to be unzipped (1.3 GB zipped).

I ran this on an iMac (2.7 GHz intel Core i5; 8 GB memory; OSX 10.10.5) with Matlab R2015b. It took several days to get through all Trinity objects. You don’t have to do this. I saved the result and placed the output in ‘completed_run_output/full_tga_osx_20151022.mat’.

This .mat contains a structure that can be opened in Matlab. This structure lists all ‘connected_islands’ for each Trinity object. The connected island borders are used in the analysis (described in the main text and elsewhere in the supplement).

To generate a Perl-compatible hash with information about connected islands, I first ran

write_island_borders.m (tga = structure loaded from ‘full_tga_osx_20151022.mat’)

then

structure_info2hash.pl

The hash generated has key = Trinity id (e.g. ‘comp7421_c0_seq1’) and the value is a hash with various info about the Trinity object:

Island_borders: start/stop for connected islands in Trinity coordinates (1 = first bp in Trinity sequence).

Minus/plus splice positions: splices detected by Matlab in Trinity coordinates.

Minus/plus splice scores: scores calculated for splices

Volvox blastx hit: start/stop for Blastx hit to Volvox in Trinity coordinates

The hash is used by ‘myNisland_accounting.pl (described elsewhere in the supplement).

Example

$VAR59 = 'comp7421_c0_seq1';

$VAR60 = {

'island_borders' => {

'1' => [

'1',

'463'

]

},

'minus_splice_scores' => [

'0',

'0',

'0'

],

'plus_splice_positions' => [

'125',

'257',

'438'

],

'Volvox_hit' => {

'Volvox_id' => 'Vocar20001420m',

'Volvox_hsp_range' => [

'15 341 '

]

},

'minus_splice_positions' => [

'0',

'0',

'0'

],

'plus_splice_scores' => [

'15',

'15',

'15'

]

};
